# Author’s reply: Vertical synergistic divergence: A distinct separate entity

**Published:** 2010

**Authors:** Jitendra Jethani

**Affiliations:** Pediatric Ophthalmology and Strabismus Clinic, Dr. Thakorbhai V Patel Eye Institute, Haribhakti Complex, Salatwada, Vadodara – 390 001, Gujarat, India

Dear Editor,

I read the comments given by Pandey *et al*.[1] It is difficult to understand how they could convert this case[2] into a bilateral Brown’s syndrome (BS) case. The only finding that bilateral superior oblique overaction is present does not point to a bilateral BS. The child on elevation of the left eye showed a direct depression of the right eye [Fig. 1]. There is a limitation of the right eye in depression. Why should that happen in a bilateral BS? The lid retraction was present in downgaze[2] and not in adduction, very similar to one seen in pseudo-Von Graefe’s sign. The adduction, depression in abduction, and elevation in adduction were restricted in the right eye but not in the left.[2] A right eye BS would not have the limitation of depression in abduction as was seen in our case.[2] The ductions were completely normal in the left eye and so a question of BS in the left eye does not arise. No Marcus Gunn jaw wink was seen which was supposedly completing the jigsaw puzzle for the authors. A forced duction test was not overlooked but the child was not cooperative and his parents did not consent for general anesthesia.[2] Not everything can be written about the case (which was discussed with the reviewers) because of the word limit.

**Figure 1:**
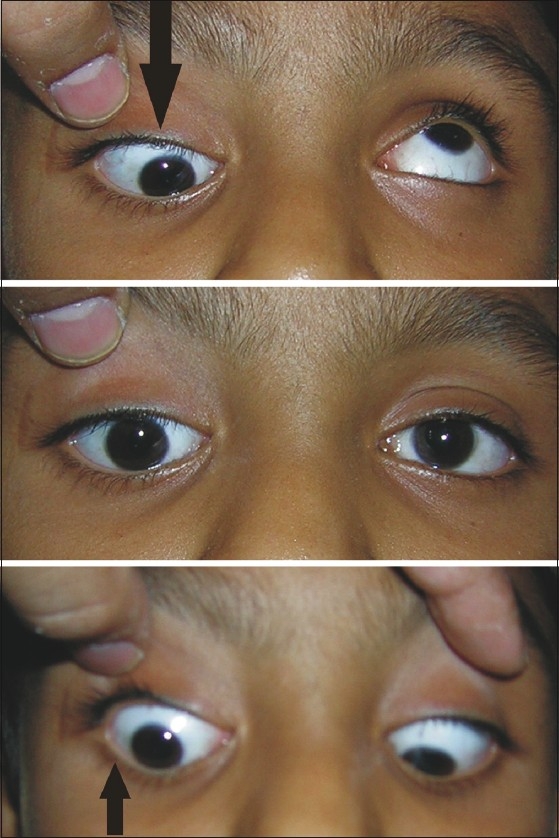
The picture clearly demonstrates the restricted depression and eyeball moving downward in the vertical gaze

The authors have wasted time and space by writing more and more about bilateral BS which was not necessary.[1] I have put up my explanation which happens to be contrary to the opinion of Pandey *et al*.[1] In science, one should respect other’s opinion and I would just respect their opinion.

## References

[1] Pandey PK, Dadeya S, Singh A, Vats P, Rathi N (2010). Misinnervation in 3^rd^ nerve palsy: Vertical synergistic divergence or consummate congenital bilateral asymmetrical Brown’s syndrome with congenital ptosis?. Indian J Ophthalmol.

[2] Jethani J (2009). Misinnervation in third nerve palsy: Vertical synergistic divergence. Indian J Ophthalmol.

